# Zika Virus Transmission from French Polynesia to Brazil

**DOI:** 10.3201/eid2110.151125

**Published:** 2015-10

**Authors:** Didier Musso

**Affiliations:** Institut Louis Malardé, Papeete, Tahiti, French Polynesia

**Keywords:** Zika virus, ZIKV, arbovirus, viruses, vector-borne infections, mosquito, *Aedes* spp., *Ae*. *africanus*, *Ae*. *luteocephalus*, *Ae*. *hensilli*, *Ae*. a*egypti*, Brazil, French Polynesia, Pacific Islands, New Caledonia, Cook Islands, Easter Island, Vanuatu

**To the Editor:** Campos et al. (*1*) reported a Zika virus (ZIKV) outbreak in Brazil in 2015. This response adds complementary data related to the propagation of this mosquitoborne disease.

To date, the largest ZIKV outbreak occurred in French Polynesia during 2013–2014. The outbreak spread to other Pacific Islands: New Caledonia, Cook Islands, Easter Island, Vanuatu, and Solomon Islands (*2*). The origin of introduction of ZIKV to French Polynesia remains unknown; introduction of ZIKV in New Caledonia was after imported cases from French Polynesia (*3*); introduction to Easter Island was suspected to have occurred among attendees of the annual Tapati festival, including those from French Polynesia (*4*). The virus was likely transmitted to New Caledonia, Cook Islands, and Easter Island when infected travelers from French Polynesia were bitten by vectors while on the islands. Frequent travel between New Caledonia and Vanuatu is likely related to the introduction of ZIKV in the latter country.

Phylogenetic studies showed that the closest strain to the one that emerged in Brazil was isolated from samples from case-patients in French Polynesia and spread among the Pacific Islands (*1*); both strains belong to the Asian lineage. It has been assumed that ZIKV was introduced to Brazil during a World Cup soccer competition in 2014 (*5*), although no ZIKV-endemic Pacific countries competed. However, in August 2014, the Va’a World Sprint Championship canoe race was held in Rio de Janeiro, Brazil. Four Pacific countries (French Polynesia, New Caledonia, Cook Islands, and Easter Island) in which ZIKV circulated during 2014 had teams engaged in this contest in several categories. These data combined with phylogenetic studies by Zanluca et al. (*5*) suggest that ZIKV introduction in Brazil may have been a consequence of this event. In areas where potential vectors are present, vigilance should be enhanced to detect imported cases of ZIKV, and laboratory capacity to confirm suspected ZIKV infections should be strengthened.
